# A fungus-eat-fungus world: *Digitopodium*, with particular reference to mycoparasites of the coffee leaf rust, *Hemileia vastatrix*

**DOI:** 10.1186/s43008-020-00052-w

**Published:** 2021-01-05

**Authors:** Adans A. Colmán, Harry C. Evans, Sara S. Salcedo-Sarmiento, Uwe Braun, Kifle Belachew-Bekele, Robert W. Barreto

**Affiliations:** 1 Departamento de Fitopatologia, Universidade Federal de Viçosa, Viçosa, MG 36570-900 Brazil; 2 CAB International, UK Centre, Egham, Surrey TW20 9TY UK; 3 Martin Luther University Halle, Institute of Biology, Department of Geobotany and Botanical Garden, Herbarium, Neuwerk 21, 06099 Halle (Saale), Germany; 4 Jimma Agricultural Research Center, Jimma, Oromia Region Ethiopia

**Keywords:** Classical biological control, Ethiopia, Fungicolous fungi, *Herpotrichiellaceae*, *Hyalocladosporiella*, New taxa`, Phylogenetics

## Abstract

*Digitopodium hemileiae* was described originally in 1930 as *Cladosporium hemileiae*; growing as a mycoparasite of the coffee leaf rust (CLR), *Hemileia vastatrix*, in a sample of diseased leaves of *Coffea canephora* collected in the Democratic Republic of Congo. No cultures from this material exist. More recently, the type material was re-examined and, based on morphological features, considered to be incorrectly placed in *Cladosporium.* The new genus *Digitopodium* was erected to accommodate this species. Interest in fungal antagonists of *H. vastarix*, as potential biocontrol agents of CLR, led to comprehensive surveys for mycoparasites, both in the African centre of origin of the rust, as well as in its South American exotic range. Among the rust specimens from Ethiopia, one was found to be colonized by a fungus congeneric with, and similar to, *D. hemileiae.* Pure cultures obtained from the Ethiopian material enabled a molecular study and for its phylogenetic position to be elucidated, based on DNA sequence data from the ITS and LSU regions. Molecular data showed that two members of the recently erected genus *Hyalocladosporiella* (*Herpotrichiellaceae*: *Chaetothyriales*) are congeneric with *Digitopodium* from Ethiopia and morphologically similar to both *D. hemileiae* and the two Ethiopian isolates. These isolates were found to be morphologically and genetically identical to *H. tectonae*, described previously from Brazil. Thus, species of *Hyalocladosporiella* are re-allocated to *Digitopodium* here; including *D. tectonae*, and a novel species, *D. canescens*, recently found in Brazil growing as a mycoparasite of *Puccinia thaliae*. The potential use of *D. hemileiae* and *D. tectonae* for classical biological control of CLR is discussed*.*

## INTRODUCTION

*Hemileia vastatrix* is the most important pathogen of coffee plants worldwide, causing coffee leaf rust (CLR) (Zambolim 2016, Talhinhas et al., 2017). The economic and social crisis provoked by CLR outbreaks of the past are well documented (Avelino et al. 2015, McCook & Vandermeer 2015). Since 2012, disastrous outbreaks of CLR have been destroying the livelihoods of the coffee growers in Central America (Avelino et al. 2015, Talhinhas et al. 2017) and have prompted mass migrations – refugee caravans – to Mexico and the USA (Ward et al. 2017).

Efforts in mitigating the impact of CLR have included a pioneering initiative towards the development of a classical biological control management strategy, based on the use of fungal natural enemies from the native range of coffee and *Hemileia vastatrix* in Africa. A number of mycoparasitic fungi of CLR have been reported previously (Carrion & Rico-Gray 2002, James et al. 2016). However, the latter records are all from the Americas, where coffee and *H. vastatrix* are exotic species. Such mycoparasites are interpreted, therefore, as generalists that have jumped from other fungal hosts and did not co-evolve as specialized parasites of the CLR fungus. Only two mycoparasites have been reported exclusively from the centre of origin of cultivated *Coffea* in Africa, namely: *Digitopodium hemileiae* (Steyaert 1930, Heuchert et al. 2005) and *Paranectriella hemileiae* (Pirozynski 1977). In order for any classical biocontrol agent to be introduced against its target in an exotic situation, it is critical to have its taxonomy fully elucidated (Scott 1995). This publication deals with a reappraisal of the taxonomy of *D. hemileiae* and related taxa, based on newly-collected specimens obtained during surveys for mycoparasites of *H. vastatrix* in Africa and of related material collected in Brazil.

## MATERIAL AND METHODS

Surveys involved scientists from Ethiopia, Brazil, and the UK and were concentrated in areas where *Coffea arabica* still occurs in the wild or is cultivated in semi-wild conditions, as in Ethiopia. At each selected site, coffee plants were examined for rust pustules – with particular attention to collecting rust colonies overgrown by other fungi, or appearing to be abnormal (unusual colour, poor sporulation). Specimens were dried in a plant press for later processing in the laboratory (preliminary identification and isolation). The dried samples were processed within 2 weeks of collection after transport to laboratories in the UK or Brazil. Mono-conidial cultures were obtained by direct isolation of the fungi by aseptic transfer of fungal propagules from colonized tissue with a sterile fine point needle onto potato dextrose-agar (PDA) plates. Pure cultures were preserved temporarily in potato carrot-agar (PCA) slants and long-term preservation was in silica-gel and in 10% glycerol at -80 °C, as described in Dhingra & Sinclair (1995). Pure cultures were deposited in the culture collection of the Universidade Federal de Viçosa (COAD) and dried specimens were deposited in the herbarium of the Universidade Federal de Viçosa (VIC).

Culture characteristics were described based on colonies formed on 2% malt extract-agar (MEA), PDA, and oatmeal-agar (OA) for 7 d at 25 ± 2 °C under a 12 h light regime (light provided by two white and one near-UV lamps placed 35 cm above the plates). Colony colour terminology followed Rayner (1970).

Morphology was described based on the structures formed on colonized rust pustules on dried specimens, complemented with observations made on slide cultures, as described in Waller et al. (1998); colonies being formed on blocks of synthetic nutrient poor-agar (SNA) (Nirenberg, 1981) for 14 d, under the conditions mentioned above. Slide cultures and fungal structures obtained directly from rust pustules were mounted in lactoglycerol or lactofuchsin and the microscope slides were examined under a light microscope, Olympus BX 53 (Olympus, Melville, NY, USA), connected to an Olympus Q-color 5 camera (Olympus, Center Valley, PA, USA). Conidial morphology was based on shape, colour, and presence or absence of septation. Biometric data were generated from the observation of at least 30 structures.

DNA was extracted from single spore isolates cultivated on potato dextrose liquid medium at 25 °C for 5 d. Total genomic DNA was extracted from approximately 50–80 mg of mycelium. Mycelial masses were disrupted with a L-Beader 3 (Loccus Biotecnologia, Cotia, SP, Brazil) adjusted to a speed of 4000 rpm, 2 cycles of 10 s each. DNA extraction was carried using the Wizard Genomic DNA Purification Kit (Promega, Madison,WI, USA), according to the manufacturer’s recommendations.

DNA PCR amplifications were performed with the primer pairs LR0R/LR5 (Vilgalys & Hester 1990) and ITS4/ITS5 (White et al. 1990) for the partial *28S* rDNA (LSU) and *ITS*/5.8 nr-DNA (ITS) regions. The polymerase chain reactions (PCR) were performed using a total volume of 12 μL in reactions with mixture containing 30 μg DNA, 0.5 μm of each primer and 1X Master mix DreamTaq DNA polymerase, as recommended by the manufacturer (Thermo Fisher Scientific Baltics, Vilnius, Lithuania). The amplification was performed for LSU with an initial denaturing at 94 °C at 5 min, followed by 40 cycles of denaturation at 94 °C for 30 s, annealing at 55 °C for 30 s, extension initial at 72 °C for 30 s, and 7 min final extension at 72 °C. The PCR products were purified by using an ExoSAP-IT purification kit (Amersham Biosciences, Arlington Heights, IL, USA), according to the manufacturer’s recommendations. Amplified fragments were sequenced by Macrogen (Seoul, South Korea, http://www.macrogen.com).

### Phylogenetic analyses

The nucleotide sequences obtained from forward and reverse primers were used to obtain consensus sequences using SeqAssem (SequentiX—Digital DNA Processing, Klein Raden, Germany) (Hepperle, 2004). Complementary sequences used in the analyses were obtained from GenBank (http:// www.ncbi.nlm.nih.gov) (Table 1). The alignment performed using MUSCLE implemented in the MEGA 7 (Kumar et al. 2016). The aligned sequences were manually corrected where needed. The consensus sequences were deposited in GenBank (Table 1) and taxonomic novelties in MycoBank (Crous et al. 2004).

**Table 1:** Isolates included in the phylogenetic analyses. GenBank numbers in boldface indicate new sequences

Species	Isolates	Substrate	Genbank number	
			LSU	ITS
*Cladosporium adianticola*	CBS 582.92	*Adiantum tenerum*	DQ008143	–
*Cladosporium adianticola*	CBS 735.87	*Adiantum* sp.	DQ008144	DQ008125
*Cladosporium cladosporioides*	CBC 109501	Deep mycosis ofhuman patient	DQ008146	–
*Cladosporium uredinicola*	CBS 306.84	*Puccinia allii*	DQ008147	–
*Digitopodium cannae* (syn. *H. cannae*)		*Puccinia thaliae* (on *Canna indica*)	–	MF072396
*Digitopodium canescens*	COAD 2928	*P. thaliae*	**MK829192**	–
*D.tectonae* (syn*. H. tectonae*	CBS 137989	*Tectona grandis*	KJ869199	KJ869142
*D. tectonae* (syn*. H. tectona*e	COAD 2639	*Olivea tectonae*	**MK829188**	**MK829191**
*D. tectonae*	COAD 2641	*Hemileia vastatrix*	**MK829193**	**MK829189**
*D. tectonae*	COAD 2640	*H. vastatrix*	**MK829190**	**MK829187**
*Metulocladosporiella musae*	CBS 161.74	*Musa* sp.	DQ008161	DQ008137
*Metulocladosporiella musae*	CBS 113863	*M. sapientum*	DQ008138	DQ008162
*Metulocladosporiella musicola*	CBS 194.63	*Musa* sp.	DQ008152	DQ008126
*Metulocladosporiella musicola*	CBS 113873	*M. sapientum*	DQ008159	DQ008152
*Metulocladosporiella musicola*	CBS 113861	*M. sapientum*	DQ008156	DQ008131
*Rhinocladiella anceps*	CBS 157.54	*Fagus sylvatica*	EU041861	EU041804
*Rhinocladiella anceps*	CBS 181.65	Soil	EU041862	EU041805
*Rhinocladiella fasciculata*	CBS 132.86	Decayed wood	EU041864	EU041807
*Rhinocladiella mackenziei*	CBS 368.92	*Homo sapiens*	EU041866	EU041809
*Rhinocladiella mackenziei*	CBS 367.92	*Homo sapiens*	EU041865	EU041808
*Rhinocladiella mackenziei*	CBS 102590	*Homo sapiens*	EU041867	EU041810
*Veronaea botryosa*	CBS 121.92	*Xanthorrhoea preissii*	EU041872	EU041815
*Veronaea botryosa*	CBS 350.65	Goat dung	MH870245	MH858603
*Veronaea botryosa*	CBS 254.57	Sansa olive slag	MH869255	MH857711
*Veronaea compacta*	CBS 268.75	Soil	MH872652	MH860917
*Veronaea japonica*	CBS 776.83	dead bamboo culm	EU041875	EU041818

Phylogenetic analyses were reconstructed by means of methods based on an analysis of Bayesian Inference (BI) of the combined LSU/ITS alignments using the Markov chain Monte Carlo (MCMC) algorithm. Models of nucleotide substitution for each gene region were determined using jModeltest 2.1.7 (Darriba et al. 2012). The likelihood values were calculated and the models were selected according to the Akaike Information Criterion (AIC). The BI analysis was completed with Mr. Bayes v.3.2.6 (Ronquist et al. 2012). Simulations were carried out with 10 million random generations and samples of tree were taken every 1000 generations. The first 2500 trees were discarded from the analysis, resulting in 10,000 trees. The phylogenetic analysis of the concatenated alignment was performed on the CIPRES web portal (Miller et al. 2010). The phylogenetic tree was viewed and edited with Figtree v 1.4.3 (Rambaut, 2016). Sequence alignments were deposited in TreeBASE – Study S27398.

## RESULTS

**Fig. 1:**
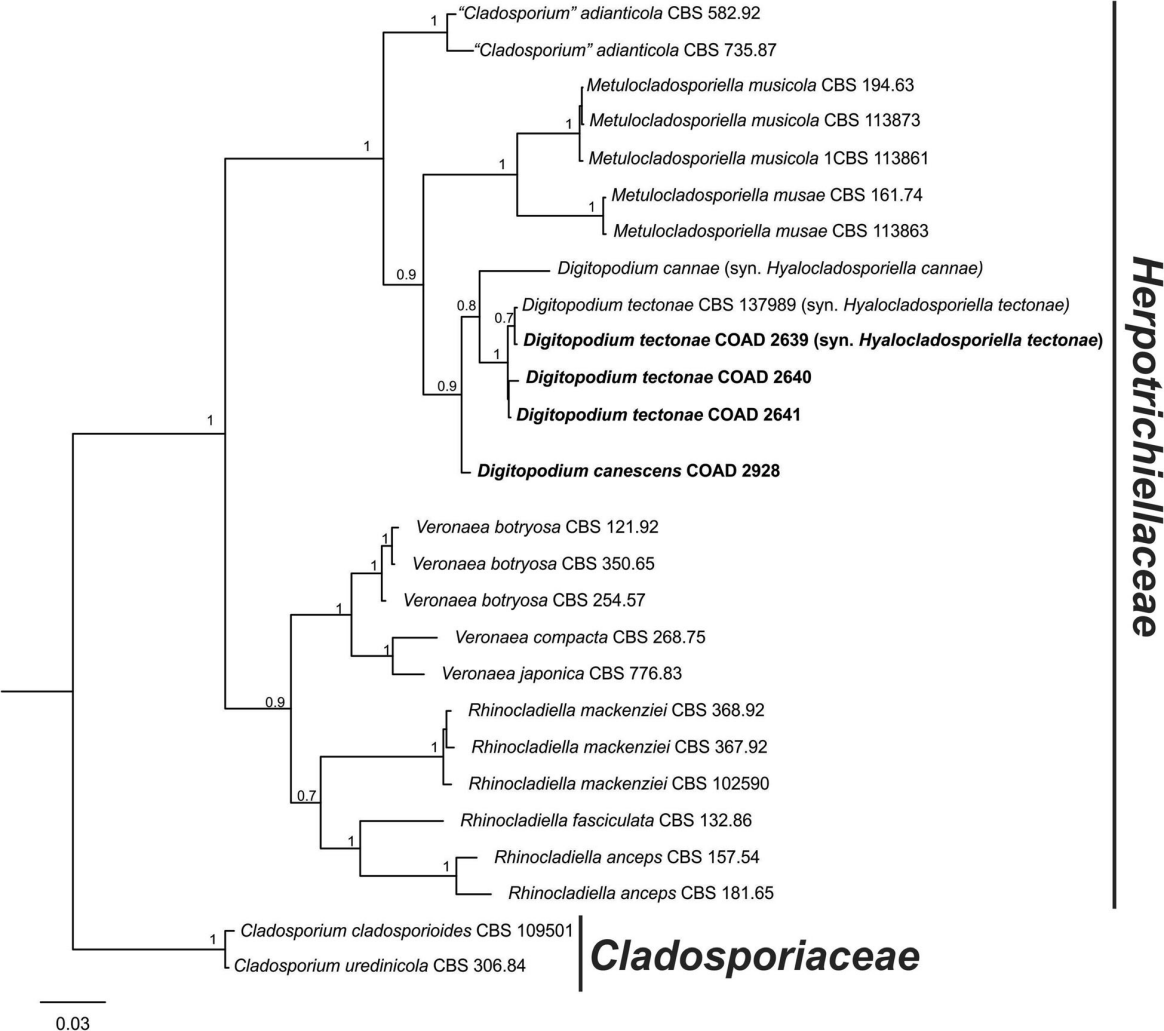
Phylogenetic study of *Digitopodium tectonae* and other *Digitopodium* isolates. Tree inferred from Bayesian analysis based on concatenated LSU and ITS sequences. Species of this study are shown in bold face. Bayesian posterior probabilities above 0.6 are indicated at the nodes. The tree was rooted with *Cladosporium cladosporioides* and *C. uredinicola* (isolates CBS 109501 and CBS 306.84)

Phylogenetic relationships were inferred using combined ITS and LSU sequences. Bayesian Inference analysis (BI) was performed combining ITS and LSU loci for the *Digitopodium* isolates and selected taxa, plus two isolates serving as outgroups (*Cladosporium uredinicola* and *C. cladosporioides*), totalling 27 isolates (Table 1). The phylogenetic analysis indicated that members now assigned to *Digitopodium* do not belong in the *Cladosporiaceae*. Instead, they form a clade together with isolates of *Hyalocladosporiella* spp. (*Chaetothyriales* – *Herpotrichiellaceae*) (Fig. 1). Sequences for isolates COAD 2928, COAD 2639, COAD 2640 and COAD 2641 were generated and included in the study.

## TAXONOMY

A comparison of the morphology of the fungus in the Ethiopian specimen showed that its general characters fitted within the morphological description given for the genus in Heuchert et al. (2005), based on the re-examination of the type material of *Cladosporium hemileiae*. The combination of the morphological evidence and the results of the molecular analysis (Fig. 1), led to the recognition that *Hyalocladosporiella* represents a younger, heterotypic synonym for *Digitopodium*, although differences in the characteristics of the conidia in the Ethiopian collection to the type of *D. hemileiae* indicated that these represent a separate species. Morphologically and genetically, the Ethiopian material is closest to *Hyalocladosporiella tectonae*, which is now transferred to *Digitopodium*, and, provisionally, maintained as a separate species, pending new collections of *D. hemileiae* suitable for epitypification, and once its phylogenetic characterization is available.

**Digitopodium** U. Braun et al., *Schlechtendalia* **13**: 65 (2005)

*Synonym*: *Hyalocladosporiella* Crous & Alfenas, *Persoonia* **32**: 237 (2014).

*Description*: *Mycelium* branched, septate hyaline, smooth. *Conidiophores* uniform or dimorphic, solitary or in loose groups. *Microconidiophores* (when present) erect, subcylindrical, straight to geniculate-sinuous, septate, brown to olivaceous brown, smooth. *Macroconidiophores* erect to slightly curved, pluriseptate, pale to dark brown, smooth, cylindrical, flexuous, unbranched or sometimes branched, either with well-developed basal digitate rhizoids or poorly developed or even absent. *Conidiogenous cells* holoblastic, integrated, terminal, subcylindrical, proliferating sympodially, but geniculations mostly not evident, smooth, brown. *Conidiogenous loci* subdenticulate, slightly thickened and darkened, not coronate. *Conidia* formed in simple or branched chains, 0–3-septate. *Ramoconidia* lacking or present. *Primary ramoconidia* (when present) fusoid-ellipsoidal to subcylindrical, septate, hila somewhat thickened and darkened, not coronate, guttulate, hyaline to pale olivaceous, smooth. *Secondary ramoconidia* (when present) in branched acropetal chains, fusoid-ellipsoidal, septate, with 1–3 apical loci somewhat thickened and darkened, not coronate, guttulate, hyaline, smooth. *Intermediary conidia* fusoid-ellipsoid, sometimes septate, guttulate, hyaline or brownish, smooth, hila almost unthickened or somewhat thickened and darkened-refractive, not coronate. *Terminal conidia* fusoid-ellipsoid, aseptate, hyaline to brownish, smooth, hilum almost unthickened or slightly thickened and darkened-refractive, not coronate.

*Type species: Digitopodium hemileiae* (Steyaert) U. Braun et al. 2005

*Note*: *Hyalocladosporiella* Crous & Alfenas is placed as a synonym of *Digitopodium* for the first time.

**Digitopodium hemileiae** (Steyaert) U. Braun, Heuchert & K. Schub., *Schlechtendalia* **13**: 66 (2005)

*Basionym*: *Cladosporium hemileiae* Steyaert, *Bull. Soc. Roy. Bot. Belgique* **63**: 47 (1930).

*Type*: **Democratic Republic of Congo** (formerly Zaire): Prov. Orientale, Biaro, near Kisangani (Stanleyville), on pustules of *Hemileia vastatrix* (*Pucciniales*) on *Coffea canephora*, Oct. 1929, *R.L. Steyaert* (BPI 426854 – holotype).

*Description*: see Heuchert et al. (2005) for a complete description.

*Notes***:** Heuchert et al. (2005) examined the type material of *Cladosporium hemileiae* and published a comprehensive description and illustration based on the holotype. *Digitopodium hemileiae* and *Hyalocladosporiella tectonae* are morphologically very close, but there are some obvious differences in the formation, pigmentation, and width of the conidia, which clearly places them into separate species. *Digitopodium hemileiae* is characterized by having conidia formed in simple chains (primary and secondary ramoconidia lacking), consistently pigmented (not hyaline), and 5–7 μm wide [vs conidia formed in branched chains (primary and secondary ramoconidia present), secondary ramoconidia, intermediary and terminal conidia colourless, much narrower, 2–3.5 μm wide]. The values of the conidial widths of *Digitopodium hemileiae* and *D. tectonae* do not even overlap.

**Fig. 2:**
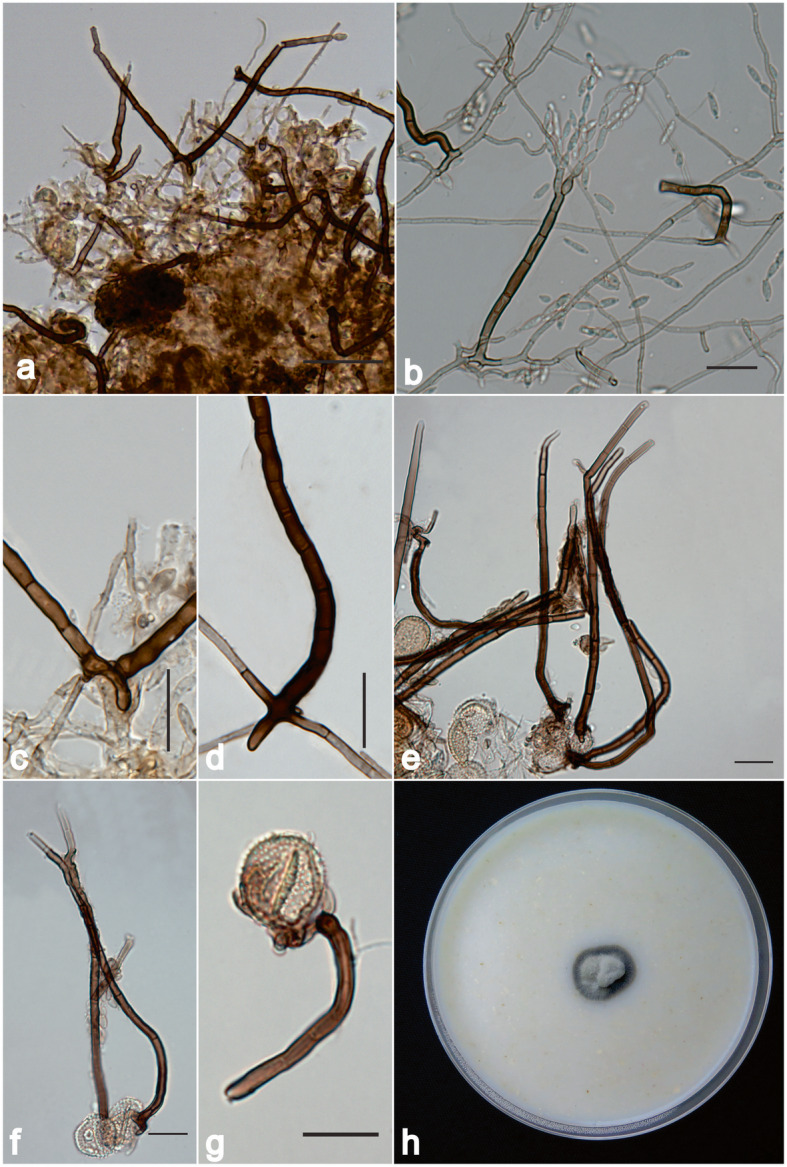
*Digitopodium tectonae* on *Hemileia vastatrix* from Ethiopia (VIC 47361; living culture COAD 2640) and *Olivea tectonae* (VIC 47183; living culture COAD 2639) from Brazil. **a** Conidiophores growing over *H. vastatrix* urediniospores. **b** Conidiophore bearing conidial chain. **c** Detail of rhizoid on *H. vastatrix* urediniospores. **d** Digitate rhizoid formed on a slide-culture. **e** Conidiophores formed on *O. tectonae* urediniospores. **f**-**g** Detail of rhizoids on *O. tectonae*. **h** Colony of COAD 2640 on oatmeal-agar. Bars = 20 μm

**Digitopodium tectonae** (Crous & Alfenas) A. Colmán & R. W. Barreto, **comb. nov. (**Fig. 2)

MycoBank, MB 832330

*Basionym*: *Hyalocladosporiella tectonae* Crous & Alfenas, *Persoonia* **32**: 237 (2014).

*Description*: Mycoparasitic on pustules of rust fungi*. Colonies* hypophyllous, on uredinia of rust pustules, producing abundant conidiophores and conidia, imparting a brownish to greyish brown (soiled) appearance to the rust sori. *Mycelium* partly immersed in pustules and intertwined with urediniospores, but mainly superficial, branched, 2–4 μm wide, septate, smooth, olivaceous to pale brown, walls thickened. *Conidiophores* solitary or in loose groups, arising from superficial hyphae or directly from colonized urediniospores, straight to slightly curved, occasionally branched, 175 (− 200) × 3–5 μm, septate, dark brown, smooth, enlarged at the base to 14 μm, with digitate/rhizoidal outgrowths, 2–16 × 2–5 μm (in vitro). *Conidiogenous cells* holoblastic, polyblastic, integrated, terminal, subcylindrical, 15–23 × 3–3.5 μm, tips somewhat curved, with up to three inconspicuous lateral conidiogenous loci, slightly thickened, slightly darkened, 0.5–1 μm diam. *Conidia* in branched, acropetal chains. *Primary ramoconidia* fusoid-ellipsoidal to subcylindrical, 8–14 × 3–3.5 μm, 1–septate, guttulate, hyaline to pale olivaceous, smooth, hila 1–3 per conidium, slightly thickened and darkened, 1 μm diam. *Secondary ramoconidia* in branched chains, fusoid-ellipsoidal, 20–26 × 3–3.5 μm, guttulate, hyaline, septate, smooth, with a single basal hilum plus 1–3 apical hila slightly thickened and darkened, 1 μm diam. *Intermediary conidia* fusoid-ellipsoid, 6–12 (− 20) × 2.5–3.5 μm, aseptate, guttulate, hyaline, smooth, hila 2 per conidium, slightly thickened and darkened. *Terminal conidia* limoniform, fusoid-ellipsoid, guttulate, 5–9 × 2.5–3 μm, aseptate, hyaline, smooth, thin-walled, hila slightly thickened, 0.5–1 μm diam.

*Morphology of structures formed* in vitro (slide cultures): *Conidiophores* 150–310 × 3.5–7.5 μm, septate, with digitate/rhizoidal base, 2–16 × 2–5 μm; *conidiogenous cells* 14–33 × 3–3.5 μm. *Primary ramoconidia* 25–30 × 3–3.5 μm, 1–3-septate, plus1–3 hila. *Secondary ramoconidia* 20–26 × 3–3.5 μm, septate, with 1–4 hila. *Intermediary conidia* (10–)12–16 (− 20) × 2.5–3.5 μm, aseptate, with 2 hila. *Terminal conidia* 12–14× 2–3 μm, aseptate, with one hilum.

*Culture characteristics*: Slow growing (12–18 mm diam after 7 d, at 25 °C), edge entire, low convex to umbonate, aerial mycelium either sparse or dense, either felty, cottony or floccose, whitish to smoke-grey to grey olivaceous, reverse grey olivaceous to dark grey olivaceous; sporulation abundant (OA), scarce (PDA), to absent (MEA).

*Type*: **Brazil**: Mato Grosso, Verde Novo, Colider, on leaves of *Tectona grandis* (*Lamiaceae*), Apr. 2013, *A.C. Alfenas* (CBS H-21702 – holotype; CBS 137989 – ex-type living culture; ITS sequence GenBank KJ869142; LSU sequence GenBank KJ869199.

*Notes*: *Digitopodium hemileiae* was originally described as *Cladosporium hemileiae* by Steyaert (1930). Much later this taxon was included in a revision of fungicolous *Cladosporium* species and redescribed as belonging to a newly erected genus *Digitopodium* by Heuchert et al. (2005), based on the feature of it having some of its conidiophores bearing distinct short digitate rhizoids at the base and lacking cladosporioid (coronate) conidial scars. During the field survey in Ethiopia, fresh material of *Digitopodium* on *H. vastatrix* pustules was collected. When compared with the description given in Heuchert et al. (2005), it was found that the morphology of the fungus from Ethiopia was similar to that described for *D. hemileiae*. Nevertheless, *D. hemileiae* lacks ramoconidia and produces broader conidia than found in the Ethiopian specimen (5 – 7 μm vs 2 – 3.5 μm diam). Additionally, conidia of *D. hemileiae* are consistently pigmented, contrary to what was observed in the Ethiopian material. The morphological similarity between *D. hemileiae*, the type species of *Digitopodium* was evident, and the collection of the mycoparasite on CLR in Ethiopia, clearly indicated that the sample from Ethiopia belonged to *Digitopodium*. Moreover, a comparison of the morphological description of *Digitopodium* with *Hyalocladosporiella* (*Herpotrichiellaceae*), corroborated by the preliminary results of the molecular study, reinforced the evidence of a connection between *Hyalocladosporiella* and *Digitopodium*.

There is no mention in Crous et al. (2014) of a digitate rhizoid base on the conidiophores of *H. tectonae*. This species was described simply as associated with teak, *Tectona grandis* (*Lamiaceae*), based on a specimen collected in the state of Mato Grosso (Brazil). An attempt to recollect type material was successfully undertaken in Feb. 2019 by R. Alfenas (pers. comm.). While processing the material, it became evident that the fungus was not directly associated with the teak plant but in fact occurs as a mycoparasite of the teak rust fungus *Olivea tectonae.* Based on these results, we consider *Hyalocladosporiella* to be a younger, heterotypic synonym of *Digitopodium*, although appropriate new collections for epitypification, and as source of sequence data, are not yet available*. Digitopodium tectonae* is undoubtedly not restricted to *Hemileia*, as previously assumed, and may have a broad host range within the *Pucciniales*. Although poorly documented, undoubtedly due to little attention being paid to such mycoparasitic fungi, *D. tectonae* seems to have a wide pantropical distribution; the only two existing records now being from distant locations: East Africa (Ethiopia) and South America (Brazil).

*Additional specimens examined*: **Ethiopia**: Oromia Region, Bale Mountains, Harenna Forest, Mayate Coffee Village, Jan. 2018, *K.B. Belachew & H.C. Evans*, on pustules of *Hemileia vastatrix* (*Pucciniales*) on *Coffea arabica* (*Rubiaceae*), (VIC47361); living culture COAD 2640, LSU sequence of COAD 2640 GenBank MK829190 and ITS sequence of COAD 2640 GenBank MK829187; Details as above, living culture COAD 2641, LSU sequence of COAD 2641 GenBank MK829193and ITS GenBank MK829189. **Brazil**: Mato Grosso, Varzea Grande, on pustules of *Olivea tectonae* (*Pucciniales*) on *Tectona grandis* (*Lamiaceae*), Feb. 2019, *R. Alfenas* (VIC 47183; living culture COAD 2639), LSU sequence of COAD 2639 GenBank MK829188 and ITS sequence of COAD 2639 GenBank MK829191.

**Digitopodium cannae** (T.K.A. Kumar) A. Colmán & R. W. Barreto, **comb. nov.**

MycoBank, MB 832331

*Basiony*m: *Hyalocladosporiella cannae* T.K.A. Kumar, *Persoonia* **39**: 307 (2017).

*Type*: **India**: Kerala, Kozhikode, on leaves of *Canna indica* (*Cannaceae*), 20 Aug. 2014, *T.K.A. Kumar* (CAL 1342 – holotype).

*Description*: For a complete description see Kumar (in Crous et al. 2017).

*Notes*: Based on molecular and morphological characters, *H. cannae* is reallocated to *Digitopodium* and the new combination *D. cannae* is made*.* Besides having molecular differences from *D. hemileiae, D. cannae* does not appear to have well-developed digitate rhizoids, as in *D. hemileiae* and *D. tectonae.* If it does, then these were overlooked (Crous et al. 2017). There is mention of a possible connection between the newly described fungus and *Puccinia thaliae* but with no certainty. Based on the phylogenetic analysis and the new combination made here, it seems that *Digitopodium* species are obligate mycoparasites of *Pucciniales*.

**Fig. 3:**
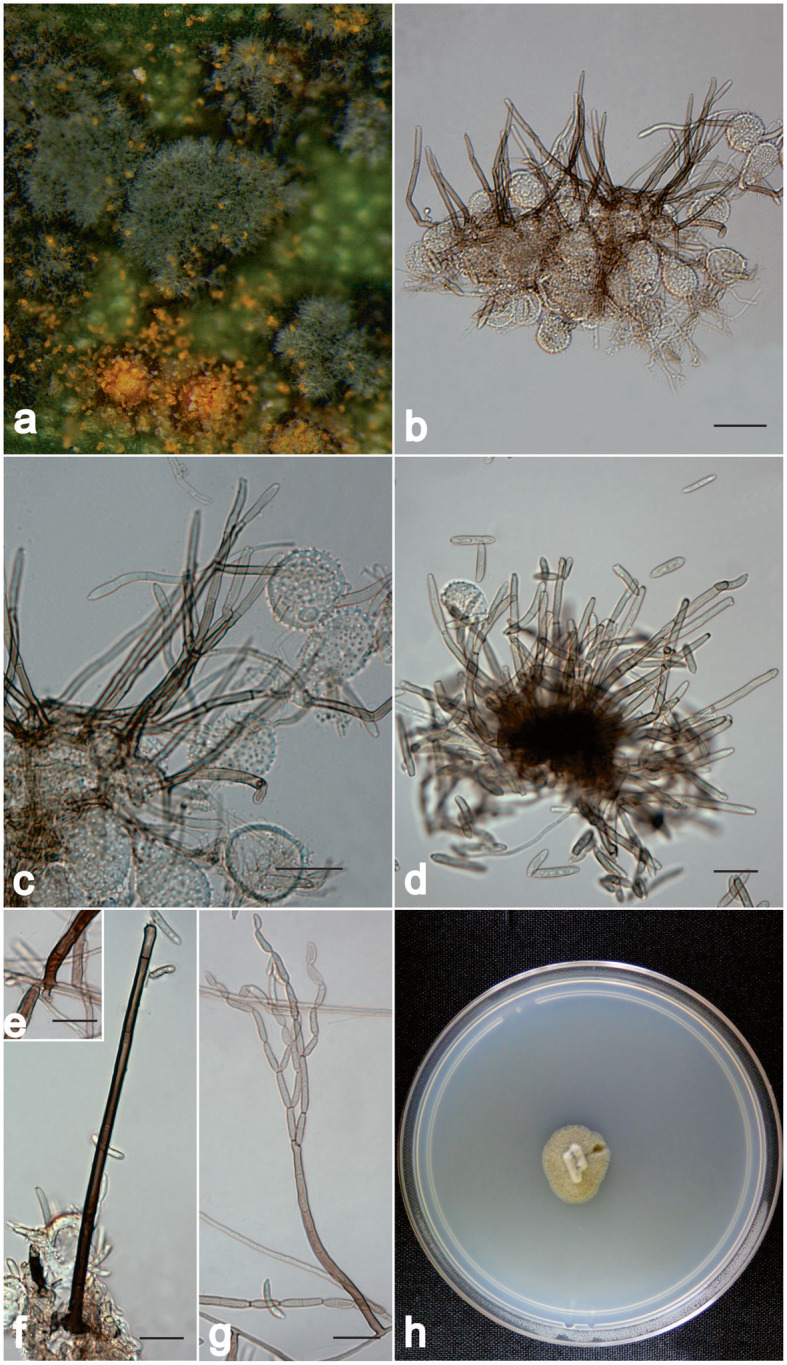
*Digitopodium canescens* on *Puccinia thaliae* from Brazil (VIC 47182; living culture COAD 2928). **a** Conidiophores formed over *P. thaliae* pustules (notice distinct grey colour). **b**-**c** Details of mycoparasitized *P. thaliae* pustules showing abundant microconidiophores of *D. canescens*. **d** Sporodochium-like aggregate of microconidiophores. **e** Detail of vestigial rhizoid at conidiophore base. **f** Macroconidiophores growing over the urediniospores. **g** Conidiophore bearing conidial chains. **h** Colony of *D. canescens* formed on synthetic nutrient-poor agar. Bars = 20 μm

**Digitopodium canescens** A. Colmán & R. W. Barreto, **sp. nov.** (Fig. 3)

MycoBank, MB 832332

*Etymology*: Named after the distinctly greyish colonies formed over the rust host pustules.

*Diagnosis*: Similar to the other species of *Digitopodium* spp. but having wider micro- and macroconidiophores than in *D. cannae*, and differing from *D. hemileiae* and *D. tectonae*, in having shorter conidiophores of two kinds – solitary (microconidiophores) or in fascicles (macroconidiophores) – and also lacking well-developed digitate rhizoids at the base of conidiophores as in *D. hemileiae* and *D. tectonae. Type*: **Brazil**, Minas Gerais, Coronel Pacheco, on rust colonies of *Puccinia thaliae* on leaves of *Canna × generalis*, 10 Dec. 2018, *R.W. Barreto* (VIC 47182 – holotype; COAD 2928 ex-type living culture; LSU sequence GenBank MK829192.

*Description: Mycelium* immersed and superficial on *Puccinia thaliae* pustules, olivaceous grey, branched, septate, 1–3 μm wide. *Conidiophores* either solitary (microconidiophores) or in loose fascicles (macroconidiophores). *Microconidiophores* erect, subcylindrical, almost straight to geniculate-sinuous, (35–) 40–74 × 3–6 μm, 1–4-septate, pale brown to olivaceous brown, smooth, thick-walled. *Conidiogenous cells* integrated, terminal, subcylindrical, 9–26 × 2.5–4 μm, somewhat thick-walled, pale brown, smooth. *Conidiogenous loci* sympodially arranged, slightly thickened and darkened. *Macroconidiophores* erect, cylindrical, flexuous, geniculate, (25) 40–140 × 3–5 μm, sparingly branched, 3–14-septate, dark brown, smooth, thick-walled, rhizoid bases present but poorly developed. *Conidiogenous cells* integrated, terminal, subcylindrical, 16–40 × 3.5–4 μm, dark brown to brown, smooth, wall slightly thickened. *Conidiogenous loci* sympodially arranged, slightly thickened and darkened. *Primary ramoconidia* ellipsoid to cylindrical, 9–34(− 40) × 3.0–4 μm, 0–2-septate, wall slightly thickened, hyaline to pale olivaceous grey; hila 2–3 per conidium, slightly thickened and darkened, smooth. *Secondary ramoconidia* in branched chains, ellipsoid to cylindrical, (11–)15–30 (− 35) × 2.5–3 μm, 0–1-septate, hyaline, smooth, guttulate, wall slightly thickened, hila 2–3 per conidium, thickened and darkened. *Intermediary conidia* fusoid-ellipsoid, 11–25 × 2.5–3 μm, 0–1-septate, guttulate, hyaline, smooth, thin-walled, hila 1–2 per conidium, slightly thickened and darkened. *Terminal conidia* limoniform to pyriform to tear-drop-shaped, ellipsoid or fusoid, (5-) 8–16 × 2.5–3 μm, aseptate, hyaline, guttulate, smooth, thin-walled, hila slightly thickened and darkened.

*Morphology of structures formed* in vitro (slide cultures): *Microconidiophores* (35–) 40–74 × 3–6 μm, 1–4-septate conidiogenous cells 9–26 × 2.5–4 μm. *Macroconidiophores* 65–180 × 2–5 μm, 3–16-septate, conidiogenous cells 8–25 × 2.5–3 μm. *Primary ramoconidia* 10–28 (− 36) × 2.5–3 μm, 0–2-septate, hila 2–3. *Secondary ramoconidia* 18–37 (− 40) × 2.5–3 μm, 0–2-septate, hila 2; intermediary conidia 15–30× 2.5–3 μm, 0–2 septate, hila 2. *Terminal conidia* 8–13 × 3–4 μm, aseptate, with one hilum.

*Culture characteristics*: Slow growing (15–18 mm diam after 12 d at 25 °C), edge entire, low convex to umbonate, aerial mycelium either sparse or dense, felted, cottony or floccose, olivaceous grey to pale olivaceous grey at periphery, olivaceous black; sporulation abundant (OA) and (PDA) to absent (MEA).

*Notes*: *Digitopodium canescens* is described here as a new species from Brazil. Morphologically it resembles *D. cannae* but can readily be distinguished by the size of its ramoconidia and conidiophores. In *D. canescens*, the micro- and macroconidiophores are wider than in *D. cannae. Digitopodium canescens* also differs from *D. hemileiae* and *D. tectonae* in having conidiophores of two kinds, viz. solitary (microconidiophores) or in loose fascicles (macroconidiophores), and not having well-developed digitate rhizoids at the conidiophore base. Phylogenetically, it forms a clade together with other *Digitopodium* species in the *Herpotrichiellaceae*, but is sufficiently morphologically distinct to confirm it as a separate species.

## DISCUSSION

The taxonomy of the genus *Cladosporium* (*Cladosporiaceae*) has recently undergone a major revision (Bensch et al. 2012, 2015). The genus previously encompassed more than 850 species. A broad molecular phylogenetic study – including sequences of the internal transcribed spacer regions ITS1 and ITS2, the 5.8S nrDNA, as well as partial actin and translation elongation factor 1-α gene sequences, of multiple isolates – has shown that only 169 species are true *Cladosporium* species (Bensch et al. 2012). Many of the existing names were not verified because of the lack of available cultures. Following this publication, many other isolates of *Cladosporium* have been reported from a wide range of substrates (soil, clinical and indoor samples) from around the world, increasing this number of accepted species to 234, including a number of new species (Crous et al. 2014, Bensch et al. 2012, 2015, Braun et al. 2015, Sandoval-Denis et al. 2015, Razafinarivo et al. 2016, Ma et al. 2017, Marin-Felix et al. 2017).

Unlike several other taxa that have been excluded from *Cladosporium* and re-allocated to other genera in Bensch et al. (2012), most of the fungicolous “*Cladosporium*-like” species included in the revision of Heuchert et al. (2005) were left out of that reappraisal. Among these was the monotypic genus *Digitopodium* proposed by Heuchert et al. (2005) to accommodate *C. hemileiae*. This fungus was originally collected in the Democratic Republic of Congo and recognized by Steyaert (1930) as a mycoparasite of *Hemileia vastatrix* (Steyaert 1930, Heuchert et al. 2005). The precise taxonomic and phylogenetic status of *D. hemileiae* remained uncertain because of the lack of pure cultures accompanying the type. *Digitopodium hemileiae* was regarded as differing from *Cladosporium* in having: inconspicuous or subconspicuous, non-coronate conidiogenous scars (loci) on the conidiogenous cells; conidiogenous cells which are not conspicuously sympodial (not geniculate); and the base of the conidiophores having short digitate rhizoid-like protuberances (Heuchert et al. 2005).

Appropriate collections of *D. hemileiae* from Central Africa that can be used for epitypification purposes, including cultures and retrieved sequence data for the phylogenetic characterization, are still lacking, but the striking morphological and ecological similarity between *D. hemileiae* and *Hyalocladosporiella tectonae* allowed us to consider the latter genus congeneric with *Digitopodium* and to propose the new combination *D. tectonae.* The case of *Digitopodium*–*Cladosporium* adds to the numerous examples of genetically unrelated hyphomycetes which have acquired through convergent evolution a striking morphological similarity, only revealed now, through the use of molecular tools. The morphological differences between *D. hemileiae* and *D. tectonae* in conidial proliferation, pigmentation and conidial width do not support the concept of a single species, but indicate that there are two different species occurring on CLR. This case is comparable to *D. cannae* and *D. canescens*, two closely allied, but morphologically and genetically distinct species mycoparasitic on the rust of *Canna* species, *Puccinia thaliae.*

Other mycoparasitic species of *Cladosporium* need to be recollected, isolated and reappraised in order to verify whether they also represent members of the genus *Digitopodium,* for example, the numerous collections and isolates uncritically assigned to and recorded as “*Cladosporium uredinicola*”*.* Specimens have been collected and recorded under that name as mycoparasites of several rust species, namely: *Chrysocyclus cestri* (syn. *Puccinia cestri*, Spegazzini 1912), *Cronartium quercuum* (Morgan-Jones & McKemy 1990), *P. horiana* (Sheta 1996), *P. araujiae* (Anderson et al., 2015), *P. melanocephala* (Ryan & Wilson 1981), *P. puta* (Barros et al. 1999), and *P. violae* (Traquair et al. 1984)*.* Although some *C. uredinicola* records are phylogenetically proven to be genuine species of *Cladosporium*, with coronate conidiogenous loci (Bensch et al. 2012), it is possible that some of these records of mycoparasites were based on misidentification and may pertain to *Digitopodium*. There is also a clear need to recollect, epitify and sequence *C. uredinicola*, originally described from Argentina by Spegazzini (1912).

Isolates of all species assigned to *Digitopodium*, including *Hyalocladosporiella*, form a strongly supported monophyletic clade in our phylogenetic tree (Fig. 1). It was also found that *Digitopodium* is relatively close to several species of *Metulocladosporiella* obtained from *Musa* spp. (Crous et al. 2006). Rhizoid-like structures were also described for *Metulocladosporiella* spp. Perhaps such structures may, in some instances, represent a useful morphological marker for the recognition of *Cladosporium*-like fungi that actually belong in the *Herpotrichiellaceae*.

It is surprising that among the four species of *Digitopodium* now recognized, one pair of species (*D. cannae* and *D. canescens*) was found on a single rust host, *Pucinia thaliae,* but on different continents, whereas another pair of *Digitopodium* species (*D. hemileiae* and *D. tectonae*) was found on another rust host, *H. vastatrix* in Africa, in addition to the occurrence of the latter species on *Olivea tectonae* in Brazil*.* This was also an unexpected finding and suggests that, unless there are specialized infraspecific genotypes of *D. tectonae*, there is no potential for this species to be deployed as a classical biological control agent in the Americas, since it already occurs in the New World without having ever been recorded on *H. vastatrix* nor producing any noticeable control of CLR. Of course, the records from the state of Mato Grosso may represent a recent occurrence of the mycoparasite, possibly introduced together with the exotic rust species *O. tectonae*. The latter was recorded for the first time in Brazil by Cabral et al. (2010) on the exotic timber tree *Tectona grandis.* As the state of Mato Grosso is distant from the coffee producing areas of Brazil, where *H. vastatrix* commonly occurs, the fungus may not have been able, as yet, to spread to these areas; or, perhaps, because surveys of mycoparasites of *H. vastatrix* in Brazil have to date involved only limited sampling, concentrated in the south-eastern states, *D. tectonae* may also be present on CLR but has remained undetected. For the moment, it seems appropriate to give priority to fungal antagonists of *H. vastatrix* other than *D. tectonae* as potential classical biological control agents of CLR. A rich diversity of mycoparasites of CLR, as well as endophytic mycoparasites within coffee plants, exists in Africa and thus there are alternative candidates for use in classical introductions (Rodríguez et al., 2020). Nevertheless, *D. hemileiae* is worthy of recollecting for taxonomic purposes, as well as for its potential application as a classical biological control agent within Africa.

## CONCLUSIONS

*Digitopodium* – formerly a monotypic genus accommodating the dematiaceous *Cladosporium*-like species, *D. hemileiae* – is expanded here with the description of the new species *D. cannae* and the synonymization of the genus *Hyalocladosporiella*, containing two species, with the recombination of *H. tectonae* and *H. cannae* into *Digitopodium*. The molecular appraisal of all three species for which DNA sequences were or became available placed *Digitopodium* clearly in the *Herpotrichiellaceae*.

It is conjectured that other *Cladosporium*-like fungi presently maintained in *Cladosporium* and related taxa may in fact belong to *Digitopodium*; and that particular attention should be given to species of *Cladosporium* mycoparasitic on members of the *Pucciniomycotina*. Circumstantial evidence indicates that *Digitopodium* is a specialist genus of species adapted to a mycoparasitic lifestyle on rust hosts and that there may be some degree of host specificity involved.

From our on-going evaluation of the fungi associated with coffee leaf rust, it is concluded that there exists a diverse and largely undocumented guild of mycoparasites attacking *Hemileia vastatrix*, especially in its African centre of origin, of which *Digitopodium* is a minor component (Rodríguez et al., 2020, Authors unpubl.). In general, mycoparasites have been poorly studied and, thus far, few have been exploited as biocontrol agents of plant pathogens. We posit that the mycoparasite niche will prove to be a vault of hidden fungal taxa filling part of the void between the number of species of fungi known to science and the progressively increasing estimates of total fungal species (Hawksworth & Lücking 2017). Exploring this niche should expand our knowledge of the magnitude of the fungal component of global biodiversity and, potentially, also provide novel, sustainable tools for plant-disease management.

## Data Availability

All data generated or analysed during this study are included in this published article. Materials, not involving intellectual property rights or other similar restrictions, will be available from the authors via request.

## Abbreviations

AIC: Akaike Information Criterion
BI: Bayesian Inference
CLR: Coffee leaf rust
COAD: Coleção Octávio de Almeida Drummond — oficial name for the culture collection of the Universidade Federal de Viçosa (Viçosa, state of Minas Gerais, Brazil)
MEA: Malt extract-agar
OA: Oatmeal-agar
PDA: Potato dextrose-agar
PCR: Polymerase chain reactions
SNA: Synthetic nutrient poor-agar
VIC: Herbarium Universidade Federal de Viçosa

## References

[CR1] Anderson FE, López SPS, Sánchez RM, Fuentealba CGR, Barton JW (2015). Puccinia araujiae, a promising classical biocontrol agent for moth plant in New Zealand: biology, host range and hyperparasitism by Cladosporium uredinicola. Biol Control.

[CR2] Avelino J, Cristancho M, Georgiou S (2015). The coffee rust crises in Colombia and Central America (2008–2013): impacts, plausible causes and proposed solutions. Food Security.

[CR3] Barros ST, Oliveira NT, Bastos STG, Mais LC (1999). Hyperparasistism of Cladosporium uredinicola over Puccinia puta on the host Ipomoea fistulosa. Mycologist.

[CR4] Bensch K, Braun U, Groenewald JZ, Crous PW (2012). The genus Cladosporium. Stud Mycol.

[CR5] Bensch K, Groenewald JZ, Braun U (2015). Common but different: the expanding realm of Cladosporium. Stud Mycol.

[CR6] Braun U, Crous PW, Nakashima C (2015). Cercosporoid fungi (Mycosphaerellaceae) 3. Species on monocots (Poaceae, true grasses). IMA Fungus.

[CR7] Cabral PGC, Capucho AS, Pereira OL, Maciel-Zambolim E, Freitas RL, Zambolim L (2010). First report of teak leaf rust disease caused by Olivea tectonae in Brazil. Aust Plant Dis Notes.

[CR8] Carrión G, Rico-Gray V (2002). Mycoparasites on the coffee rust in Mexico. Fungal Divers.

[CR9] Crous PW, Gams W, Stalpers JA, Robert V, Stegehuis G (2004). MycoBank: an online initiative to launch mycology into the 21st century. Stud Mycol.

[CR10] Crous PW, Schoers H-F, Groenewald JZ (2006). Metulocladosporiella gen. Nov. for the causal organism of Cladosporium speckle disease of banana. Mycol Res.

[CR11] Crous PW, Shivas RG, Quaedvlieg W (2014). Fungal planet description sheets: 214–280. Persoonia.

[CR12] Crous PW, Wingfield MJ, Burgess TI (2017). Fungal planet description sheets: 625–715. Persoonia.

[CR13] Darriba D, Taboada GL, Doallo R, Posada D (2012). jModelTest 2: more models, new heuristics and high-performance computing. Nat Methods.

[CR14] Dhingra OD, Sinclair JB (1995). Basic plant pathology methods.

[CR15] Hawksworth DL, Lücking R, Heitman J, Howlett B, Crous P, Stukenbrock E, James T, Gow N (2017). Fungal diversity revisited: 2.2 to 3.8 million species. The Fungal Kingdom.

[CR16] Hepperle D (2004). SeqAssem©. A sequence analysis tool, contig assembler and trace data visualization tool for molecular sequences.

[CR17] Heuchert B, Braun U, Schubert K (2005). Morphotaxonomic revision of fungicolous Cladosporium species (Hyphomycetes). Schlechtendalia.

[CR18] James TY, Marino JA, Perfecto I, Vandermeer J (2016). Identification of putative coffee rust mycoparasites via single-molecule DNA sequencing of infected pustules. Appl Environ Microbiol.

[CR19] Kumar S, Stecher G, Tamura K (2016). MEGA7: molecular evolutionary genetics analysis version 7.0. Mol Biol Evol.

[CR20] Ma R, Chen Q, Fan YL (2017). Six new soil-inhabiting Cladosporium species from plateaus in China. Mycologia.

[CR21] Marin-Felix Y, Groenewald JZ, Cai L (2017). Genera of phytopathogenic fungi: GOPHY 1. Stud Mycol.

[CR22] McCook S, Vandermeer J (2015). The big rust and the red queen: long-term perspectives on coffee rust research. Phytopathology.

[CR23] Miller MA, Pfeiffer W, Schwartz T (2010) Creating the CIPRES science gateway for inference of large phylogenetic trees. In: Proceedings of the gateway computing environments workshop (GCE), New Orleans, LA: IEEE. pp 1–8

[CR24] Morgan-Jones G, McKemy JM (1990). Studies in the genus Cladosporium sensu lato: I. concerning Cladosporium uredinicola, occurring on telial columns of Cronartium quercuum and other hosts. Mycotaxon.

[CR25] Nirenberg HI (1981). A simplified method for identifying Fusarium spp. occurring on wheat. Can J Bot.

[CR26] Pirozynski KA (1977). Notes on hyperparasitic Sphaeriales, Hypocreales and ‘hypocreoid Dothideales’. Kew Bull.

[CR27] Rambaut A (2016). FigTree 1.2.2.

[CR28] Rayner RW (1970). A mycological colour chart.

[CR29] Razafinarivo J, Jany JL, Crous PW (2016). Cladosporium lebrasiae, a new fungal species isolated from milk bread rolls in France. Fungal Biol.

[CR30] Rodríguez MCH, Evans HC, Abreu LM, Macedo DMM, Ndacnou MK, Bekele KB, Barreto RW (2020) New species and records of Trichoderma isolated as mycoparasites and endophytes from cultivated and wild coffee in Africa. Sci Rep (in press) 10.1038/s41598-021-84111-1 PMC7952591 33707461

[CR31] Ronquist FM, Teslenko P, van der Mark DL (2012). MrBayes 3.2: Efficient Bayesian phylogenetic inference and model choice across a large model space. Syst Biol.

[CR32] Ryan CC, Wilson JA (1981). A possible hyper-parasite of sugarcane rust – Cladosporium uredinicola Speg. Sugarcane Pathol Newsl.

[CR33] Sandoval-Denis MP, Sutton DA, Martin-Vicente A (2015). Cladosporium species recovered from clinical samples in the United States. J Clin Microbiol.

[CR34] Scott JK (1995). Classical biological control of plant pathogens. Adv Plant Pathol.

[CR35] Sheta W (1996). Detection of Cladosporium uredinicola in pustules of chrysanthemum white rust (Puccinia horiana). Plant Dis.

[CR36] Spegazzini CL (1912). Mycetes argentinenses (series vi). Anales del Museo Nacional de Historia Natural de Buenos Aires.

[CR37] Steyaert RL (1930). Cladosporium hemileiae n. spec. Un parasite de l’Hemileia vastatrix Berk. et Br. Bulla Soc R Botanique Belg.

[CR38] Talhinhas P, Batista D, Diniz I (2017). The coffee leaf rust pathogen Hemileia vastatrix: one and a half centuries around the tropics. Mol Plant Pathol.

[CR39] Traquair JA, Meloche RB, Jarvis WR, Baker KW (1984). Hyperparasitism of Puccinia violae by Cladosporium uredinicola. Can J Bot.

[CR40] Vilgalys R, Hester M (1990). Rapid genetic identification and mapping of enzymatically amplified ribosomal DNA from several Cryptococcus species. J Bacteriol.

[CR41] Waller J, Holderness M, Ritchie BJ (1998). Plant clinic handbook.

[CR42] Ward R, Gonthier D, Nicholls C (2017). Ecological resilience to coffee rust: varietal adaptations of coffee farmers in Copán, Honduras. Agroecol Sustain Food Syst.

[CR43] White TJ, Bruns T, Lee S, Taylor JW, Innis MA, Gelfand DH, Sninsky JJ, White TJ (1990). Amplification and direct sequencing of fungal ribosomal RNA genes for phylogenetics. PCR protocols: a guide to methods and applications.

[CR44] Zambolim L (2016). Current status and management of coffee leaf rust in Brazil. Trop Plant Pathol.

